# Visual evoked potentials for the evaluation of CNS impairment in type 1 diabetes in children

**DOI:** 10.1186/1687-9856-2013-S1-P11

**Published:** 2013-10-03

**Authors:** Heon-Seok Han, Jae Hong Yu

**Affiliations:** 1 Department of Pediatrics, Chungbuk National University hospital,Cheongju, Korea; 2 Joy Children’ Hospital, Daejeon, Korea

## 

Central nervous system (CNS) impairment is common in diabetic patients even in the early stage of the disease and it could be associated with peripheral neuropathy. Nevertheless, less attention has been directed toward the chronic effects of diabetes on the CNS.

The aim of the study was to investigate the changes of central nerve conduction in children with insulin-dependent diabetes mellitus prospectively using the visual evoked potentials (VEP) and to know how those results were related to clinical risk factors and parameters of peripheral nerve conduction studies (NCS).

A total of 75 diabetics (29 males) aged 5-26 years (mean 14.3±4.7) underwent VEP and NCS annually for 5 years. For comparison, 52 healthy children were studied. Out of 75 patients, 25 patients completed annual studies for 5 years. The latencies of P100 were prolonged at the study entry when compared with controls (*P*< 0.001). Significant positive correlations were found between the VEP latency and the glycosylated hemoglobin level. The values of latency and amplitude were inversely related with the age of patients and the duration of the disease. The values of latency were not related with parameters of NCS. Only a few parameters of NCS were weakly associated with the amplitudes of P100.

Poor glycemic control proved to be an important risk factor over 5 years as related to the development of subclinical central neural pathway abnormality. VEP could be considered as a valid noninvasive tool for detecting early diabetic central conduction abnormalities such as retinopathy or optic neuropathy.

